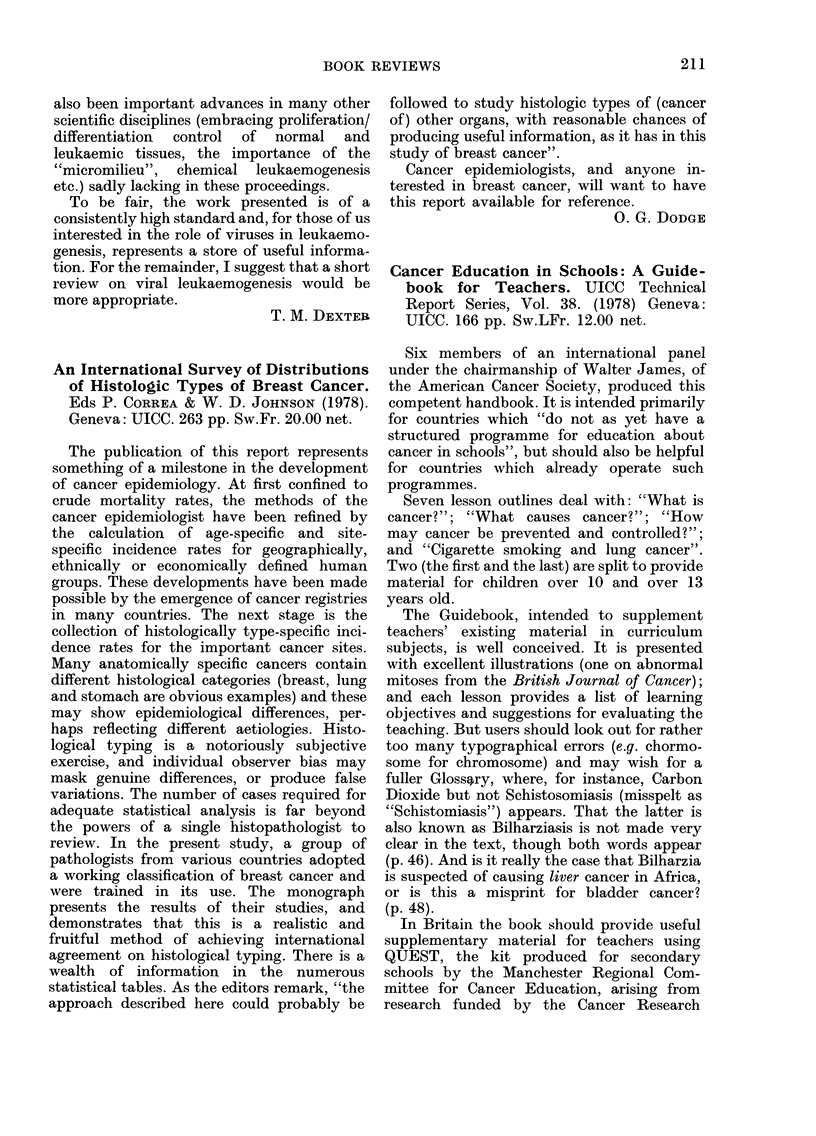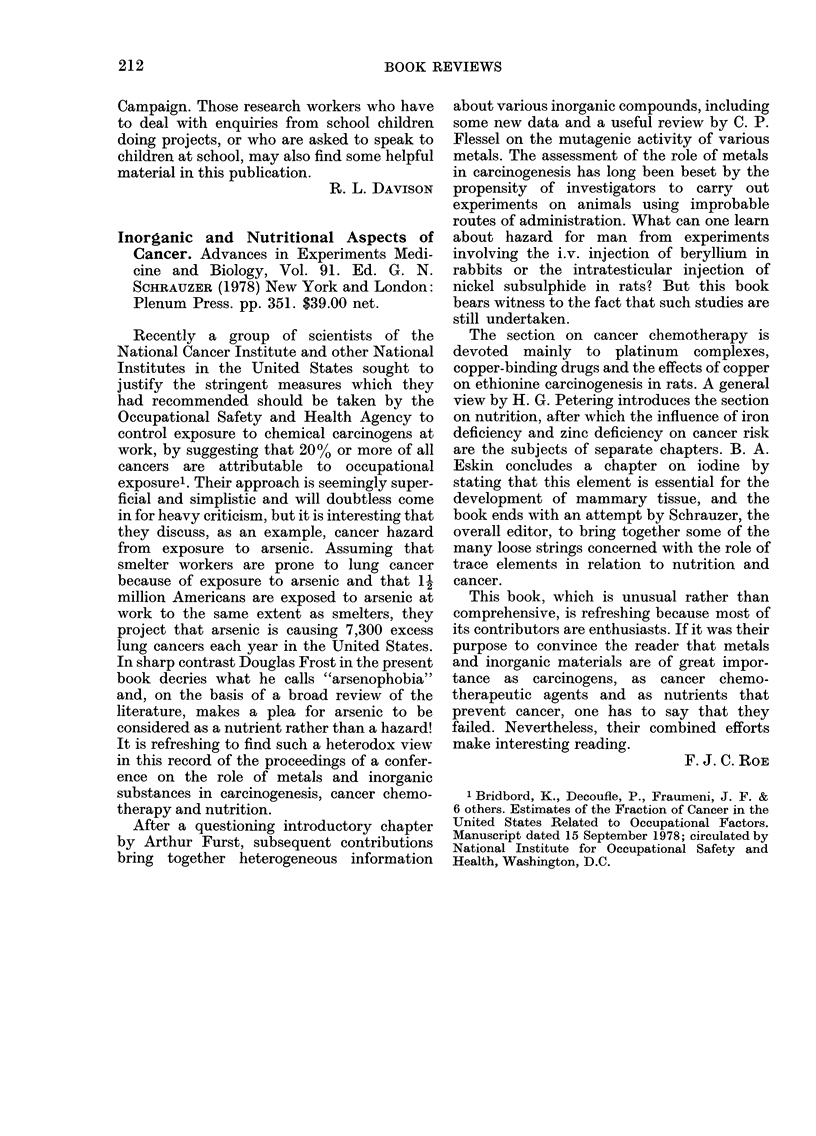# Cancer Education in Schools: A Guidebook for Teachers

**Published:** 1979-02

**Authors:** R. L. Davison


**Cancer Education in Schools: A Guidebook for Teachers.** UICC Technical Report Series, Vol. 38. (1978) Geneva: UICC. 166 pp. Sw.LFr. 12.00 net.

Six members of an international panel under the chairmanship of Walter James, of the American Cancer Society, produced this competent handbook. It is intended primarily for countries which "do not as yet have a structured programme for education about cancer in schools", but should also be helpful for countries which already operate such programmes.

Seven lesson outlines deal with: "What is cancer?"; "What causes cancer?"; "How may cancer be prevented and controlled?"; and "Cigarette smoking and lung cancer". Two (the first and the last) are split to provide material for children over 10 and over 13 years old.

The Guidebook, intended to supplement teachers' existing material in curriculum subjects, is well conceived. It is presented with excellent illustrations (one on abnormal mitoses from the *British Journal of Cancer*); and each lesson provides a list of learning objectives and suggestions for evaluating the teaching. But users should look out for rather too many typographical errors (*e.g.* chormosome for chromosome) and may wish for a fuller Glossary, where, for instance, Carbon Dioxide but not Schistosomiasis (misspelt as "Schistomiasis") appears. That the latter is also known as Bilharziasis is not made very clear in the text, though both words appear (p. 46). And is it really the case that Bilharzia is suspected of causing *liver* cancer in Africa, or is this a misprint for bladder cancer? (p. 48).

In Britain the book should provide useful supplementary material for teachers using QUEST, the kit produced for secondary schools by the Manchester Regional Committee for Cancer Education, arising from research funded by the Cancer Research

Campaign. Those research workers who have to deal with enquiries from school children doing projects, or who are asked to speak to children at school, may also find some helpful material in this publication.

R. L. DAVISON